# Magnetic resonance enterography to predict subsequent disabling Crohn’s disease in newly diagnosed patients (METRIC-EF)—multivariable prediction model, multicentre diagnostic inception cohort

**DOI:** 10.1007/s00330-025-11636-8

**Published:** 2025-05-14

**Authors:** Stuart A. Taylor, Shankar Kumar, Thomas Parry, Sue Mallett, Simon Travis, Tim Raine, Caroline Clarke, Jing Yi Weng, Gauraang Bhatnagar, Stuart Bloom, Peter John Hamlin, Ailsa Hart, Roser Vega, Maira Hameed, Anisha Bhagwanani, Rebecca Greenhalgh, Emma Helbren, James Stephenson, Ian Zealley, Vivienne Eze, Jamie Franklin, Alison Corr, Arun Gupta, Damian Tolan, William Hogg, Antony Higginson, Mohamed Ahmed, Louise Lee, Richard Pollok, Jaymin Patel, Samantha Baillie, Steve Halligan, Andrew Plumb

**Affiliations:** 1 Centre for Medical Imaging, University College London (UCL), London, UK; 2 Kennedy Institute and Translational Gastroenterology Unit, University of Oxford and Biomedical Research Centre, Oxford, UK; 3 Department of Gastroenterology, Cambridge University Hospitals NHS Foundation Trust, Cambridge, UK; 4 Research Department of Primary Care and Population Health, University College London (UCL), London, UK; 5 Department of Radiology, Frimley Park Hospital, Surrey, UK; 6 Department of Gastroenterology, University College London Hospitals NHS Foundation Trust, London, UK; 7 Department of Gastroenterology, St James’s University Hospital, Leeds Teaching Hospitals NHS Trust, Leeds, UK; 8 Inflammatory Bowel Disease Unit, St Mark’s Hospital, LNWUH NHS Trust, Harrow, UK; 9 Department of Intestinal Imaging, St Mark’s Hospital, LNWUH NHS Trust, Harrow, UK; 10 Department of Radiology, Hull University Teaching Hospitals NHS Trust, Hull, UK; 11 Department of Radiology, University Hospitals of Leicester NHS Foundation Trust, Leicester, UK; 12 Department of Radiology, Ninewells Hospital and Medical School, Dundee, UK; 13 Department of Radiology, Maidstone and Tunbridge Wells NHS Foundation Trust, Kent, UK; 14 Institute of Medical Imaging & Visualisation, Department of Medical Science & Public Health, Faculty of Health & Social Sciences, Bournemouth University, Bournemouth, UK; 15 Department of Radiology, St James’s University Hospital, Leeds Teaching Hospitals NHS Trust, Leeds, UK; 16 Department of Radiology, Queen Alexandra Hospital, Portsmouth Hospitals NHS Trust, Portsmouth, UK; 17 Infection and Immunity Research Institute, St George’s University of London, London, UK; 18 Department of Gastroenterology, St George’s University Hospitals NHS Foundation Trust London, London, UK; 19 Department of Radiology, St George’s University Hospitals NHS Foundation Trust London, London, UK

**Keywords:** Crohn’s disease, Magnetic resonance imaging, Prognostic model

## Abstract

**Objectives:**

Magnetic resonance enterography (MRE) is a first-line investigation to diagnose Crohn’s disease (CD), but its role for prognostication is unknown. Accordingly, we assessed the predictive ability of prognostic models including MRE scores (MRE Global Score (MEGS), simplified MR Index of Activity (sMARIA), and Lémann index (LI)) against models using clinical predictors alone for the development of modified Beaugerie disabling CD (MBDD) within 5 years of diagnosis.

**Methods:**

This was a multicentre, diagnostic inception cohort of patients with newly diagnosed CD across 9 UK hospitals, followed for 4 years or more. We censored development of MBDD ≤ 90 days from diagnosis, and used time-to-event models using Royston-Parmer flexible parametric models.

**Results:**

We included 194 patients, median age 29, IQR 22–44 years, 52% female. Within 5 years of diagnosis, 42% (81/194) developed MBDD. In univariable analysis, initial steroid requirement was associated with increased risk of developing MBDD (HR 2.11 (95% CI 1.36, 3.26). The baseline clinical model had 49% (39, 60) sensitivity and 66% (57, 74) specificity for predicting the top 40% of patients with the greatest risk of developing MBDD, and 86% (77, 92) sensitivity and 35% (27, 45) specificity for predicting the development of MBDD in patients with an absolute risk of ≥ 10%. There was no significant difference in sensitivity when the MEGS, sMARIA, or LI were added to the baseline clinical model.

**Conclusions:**

Addition of MRE scores at diagnosis to a multivariable model comprising clinical predictors did not improve prediction of MBDD within 5 years of diagnosis.

****Key Points**:**

***Question*** *Magnetic resonance enterography (MRE) is important for diagnosing and monitoring Crohn’s disease (CD), but primary research evaluating its prognostic role is lacking*.

***Findings*** *Adding MRE findings at diagnosis to a multivariable model comprising clinical predictors did not improve the prediction of disabling CD within 5 years of diagnosis*.

***Clinical relevance*** *When tested in a prospective, multicentre trial, current MRE activity and damage scores at diagnosis did not reliably predict whether patients would subsequently develop disabling CD. Notwithstanding this finding, MRE remains an essential tool for diagnosis and monitoring*.

**Graphical Abstract:**

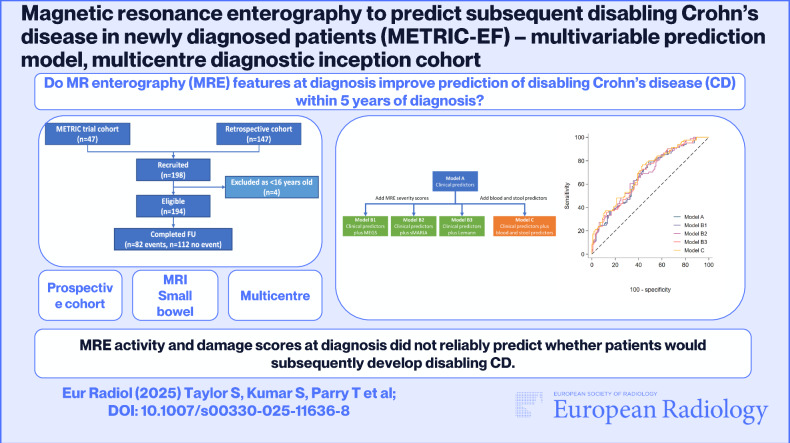

## Introduction

Crohn’s disease (CD) frequently causes considerable morbidity from intrusive symptoms and associated complications, including bowel strictures, fistulae and abscesses [1–4]. ‘Disabling disease’ implies a progressive disease course with burdening events such as the development of complications, hospitalisations, steroid dependency, the need for immunosuppression or resective bowel surgery, although it does not have a universally agreed-upon definition [5, 6]. Early treatment with biologic and immunomodulator therapy in a ‘top-down’ fashion is increasingly advocated to reduce progression to disabling disease [7–9]. However, not all patients will progress and immunomodulation is associated with side effects and is expensive [10]. Accurate prognostication would facilitate early, aggressive treatment for those most likely to benefit, while avoiding over-treatment, side-effects, complications, and costs in others, and improve outcomes overall, but such a tool is yet to be described [11].

Magnetic resonance enterography (MRE), a first-line investigation for CD, can quantify both bowel damage and underlying inflammatory activity simultaneously [12–18], but primary research evaluating its prognostic utility is sparse [19]. Although some studies have found that intestinal strictures, fistulae and abscesses on MRE are associated with increased bowel resection subsequently, no study has considered newly diagnosed patients exclusively, the group in whom prognostication would be most beneficial [20–23]. Furthermore, studies have rarely considered whether MRE can predict adverse non-surgical outcomes. Therefore, we undertook the METRIC-EF trial (Magnetic Resonance Enterography (MRE) or ulTRasound In CD extended follow-up for predicting disabling disease) to address the question: ‘Do MRE features at diagnosis improve prediction of disabling CD within 5 years of diagnosis?’

## Materials and methods

### Study design

METRIC-EF was a non-randomised, single-arm, multicentre diagnostic inception cohort of adult patients with newly diagnosed CD followed for at least 4 years.

### Study population

**Fig. 1:**
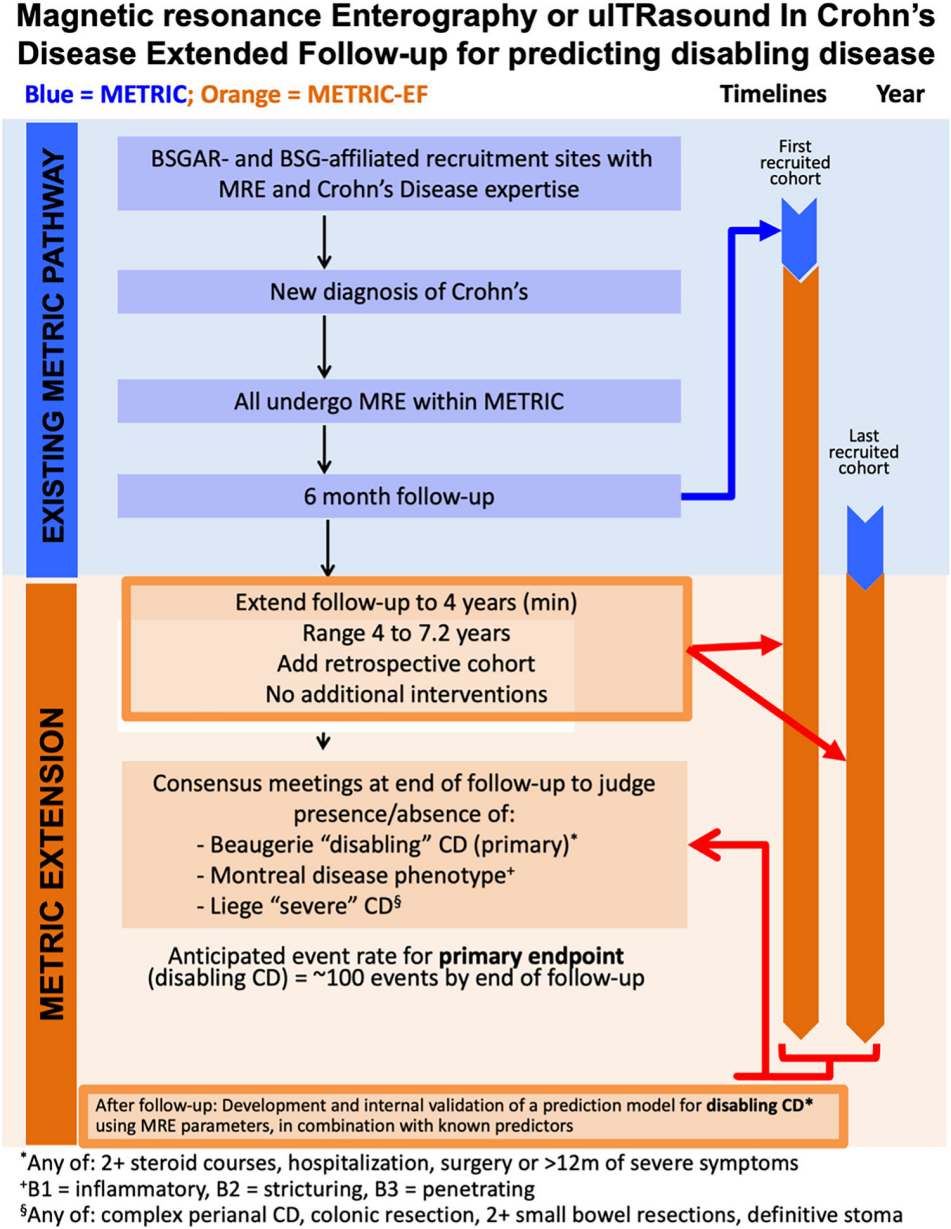
Flow diagram outlining the stages of the METRIC-EF trial. BSGAR, British Society of Gastrointestinal and Abdominal Radiology; BSG, British Society of Gastroenterology; CD, Crohn’s disease; MRE, magnetic resonance enterography; METRIC, magnetic resonance enterography or ultrasound in Crohn’s disease

METRIC (Magnetic Resonance Enterography or Ultrasound In Crohn’s Disease) was a multicentre, prospective trial performed in nine UK National Health Service (NHS) hospitals that compared diagnostic accuracy of MRE and US for the location and extent of CD [24]. Consenting adults presenting with either newly diagnosed CD or with suspected relapse were recruited. All underwent both MRE and US. For METRIC-EF, we identified those METRIC participants recruited with a new diagnosis of CD and extended their trial follow-up to a minimum of 4 years. To achieve an adequate sample size, we supplemented these participants with a carefully matched, retrospectively identified group of patients, also newly diagnosed with CD (Fig. 1). Eligibility criteria are provided in Appendix 1 and in the full trial protocol [25].

### Ethics

#### Ethical permission and consent

The METRIC-EF study achieved National Health Service research ethics committee (NHS REC), London—Hampstead Research Ethics Committee approval on 26th October, 2018 (IRAS 217422).

### Magnetic resonance imaging

#### Sequences

A standard minimum MRE sequence dataset (1.5 T or 3 T) was acquired (Appendix 2).

#### Activity and bowel damage scores

We calculated the following established MRE indices: (a) Magnetic Resonance Enterography Global Score (MEGS), (b) the simplified magnetic resonance index of activity (sMARIA, and (c) the Lémann index (LI) (Appendix 3).

#### Interpretation and blinding

MRE was interpreted by one of 11 gastrointestinal radiologists; they did not interpret cases from their own hospital and were blinded to all clinical information other than that necessary to calculate the relevant index (e.g., surgical history for LI). All readers were Consultant radiologists with subspecialty training in abdominal imaging with a minimum experience of 5 years, Fellows of the Royal College of Radiologists, and active members of the British Society of Gastrointestinal and Abdominal Radiology (BSGAR). Prior to the study commencing, the readers received a training video recorded by the lead trial radiologists, which described the scoring systems with detailed instructions and example images that defined how to score each parameter for the various scores.

### Assessment of disabling disease at follow-up

#### Follow-up

Follow-up was extended to 4 years minimum. Since participants were recruited to METRIC over 30 months, this corresponded to a mean follow-up of 5.5 years. We used this because the literature suggests clinically relevant complications of CD, i.e., disabling CD, will manifest within this time horizon [26–28].

#### Primary definition of disabling disease

Beaugerie et al [6] have previously defined ‘disabling disease’ if at least 1 of the following have occurred: more than 2 steroid courses required and/or dependence on steroids; further hospitalisation after diagnosis for flare-up or complication of the disease; presence of disabling chronic symptoms (cummulative time of more than 12 months of disabling symptoms [diarrhoea with nocturnal and/or urgent stools, intense abdominal pain because of intestinal obstruction, fever, fatigue attributable to the disease, joint pain, painful uveitis or pyoderma gangrenosum]; need for immunosuppressive therapy; and intestinal resection or surgical operation for perianal disease. We adopted a modified version of ‘disabling disease’ clarifying some symptoms and excluding the use of disease-modifying therapy as a criterion (Table 1). We also excluded patients with disabling disease at diagnosis or occurring within 90 days, since our prognostic model aimed to predict patients at risk of future disabling disease. Disabling disease was therefore defined as any of the events occurring more than 90 days after diagnosis listed in Table 1.

**Table 1:** Disabling disease-defining events if occurring more than 90 days after diagnosis

Hospitalisation for flare or complication, judged by the treating clinician.
More than 2 independent corticosteroid courses required over the follow-up period and/or dependence on corticosteroids.
Any intestinal resection > 50 cm, or surgical operation for perianal disease (examination under anaesthesia without seton placement was excluded and abscess drainage and/or seton placement included).
Chronic disabling symptoms, defined as a cumulative time of over 12 months of one or more of:
• Diarrhoea with nocturnal stool
• Anal urgency
• Abdominal pain due to intestinal obstruction (with imaging and /or surgical confirmation)
• Fever (documented tympanic temperature of > 38.0 °C or oral temperature of > 38.3 °C)
• Fatigue
• Joint pain not due to alternative causes
• Uveitis
• Pyoderma gangrenosum

#### Alternative definitions of disabling disease

We also collected the Liège [28] and Montreal criteria (Appendix 4) [29].

#### Consensus panel assessment of disease outcome and collection of model clinical predictors

Consensus panels were convened at each recruitment site, comprising, at minimum, one gastroenterologist and one radiologist, aided by the site research nurse. Panels reviewed all available clinical information over the complete follow up period and recorded the presence or absence of disabling disease. Clinical and imaging data were used by the panel to determine the Montreal classification. Clinical predictor data at diagnosis (defined a priori by the study protocol) to develop the model were also collated when available (Appendix 5). These clinical predictors were selected after a thorough literature review, and discussion with the trial Consultant Gastroenterologists, all with extensive clinical and research experience in CD [19, 26, 30].

### Outcome measures

The primary outcome was the comparative predictive ability of prognostic models incorporating MRE scores in combination with clinical predictors compared with a model incorporating clinical predictors alone, to predict the development of MBDD within 5 years of diagnosis.

Prespecified secondary outcomes were to repeat the primary outcome analysis, but defining disabling disease using the Montreal behaviour and Liège criteria instead of MBDD. We also aimed to identify which MRE parameters were most predictive, via principal component analysis (PCA), and studied whether adding the CRP, WBC count, faecal calprotectin, haemoglobin and platelet count to the baseline clinical model improved the predictive ability for MBDD.

### Powering and statistical analysis

Full details of the power calculation, statistical analysis, and prognostic model development are given in Appendix 6. In summary, we assumed the prevalence of our modified Beaugerie disabling CD (MBDD) definition of disabling disease at 5 years to be 55% to 60% [28]. We fixed the clinical predictors to be included in the multivariable models a priori since we were explicit that we would evaluate MRE scores in the context of a stable clinical model. Statistical literature at the time of study design suggested 80 to 100 events for model evaluation using prespecified and fixed predictors [31]. The sample size was based on 207 participants, of whom 114 to 124 were expected to develop MBDD.

For the primary objective, we developed a Royston-Parmer flexible parametric multivariable prognostic model [32]. We used multiple imputation for missing predictors. We developed two models based on prespecified clinical predictors (one without and one with CRP, WBC count, faecal calprotectin, haemoglobin and platelet count). We evaluated whether adding MRE scores (MEGS, sMARIA, and LI) improved the predictive ability of the base model (based on clinical predictors alone without blood and stool markers) for MBDD.

### Prognostic models

**Fig. 2:**
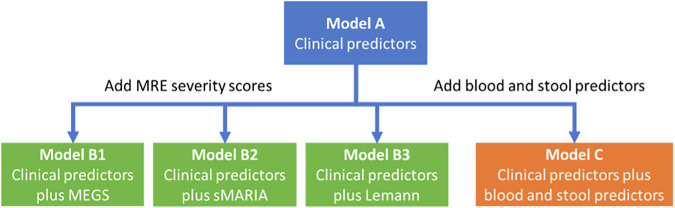
Summary of the derivation of prognostic models for developing disabling disease according to the modified Beaugerie criteria. sMARIA, simplified magnetic resonance index of severity; MEGS, Magnetic Resonance Enterography Global Score

Model A included the prespecified clinical predictors (excluding the blood and stool markers). Models B1, B2 and B3 added the MEGS, sMARIA and LI scores, respectively. Model C added the blood and stool markers to the baseline clinical model. A summary of the various models is shown in Fig. 2.

To evaluate the predictive ability of the various models in a clinically meaningful way, the trial investigators a priori predefined two risk group definitions to classify patients as either ‘high-risk’ or ‘low-risk’ for developing MBDD. High-risk group 1 (RD1) was defined as the top 40% with the greatest predicted risk. High-risk group 2 (RD2) was defined as patients with an absolute risk of developing MBDD ≥ 10%. We calculated the absolute risk cut-off by sorting patients by predicted risk and using the risk of the 8^th^ (10% of 81) patient who developed MBDD. For each risk group definition, we estimated and compared the sensitivity, specificity, and net benefit of the clinical predictor only model (model A) against the models adding MRE severity scores to the clinical predictors (models B1 (MEGS), B2 (sMARIA) and B3 (Lemann)), and when including clinical predictors as well as blood and stool markers (model C).

We performed a PCA to identify the best combination of MRE features for predicting the development of MBDD, predefining 11 features (Appendix 7) [33].

### Exploratory analyses

In a post hoc exploratory analysis, we stratified clinical and MRE predictors according to whether patients did or did not undergo resection bowel surgery within 5 years, as an alternative definition of adverse disease outcome. We also considered if starting early biologic therapy conferred any protection against developing MBDD by comparing outcomes of those who did and not start biologic therapy within 180 days of diagnosis.

## Results

### Participants

**Fig. 3:**
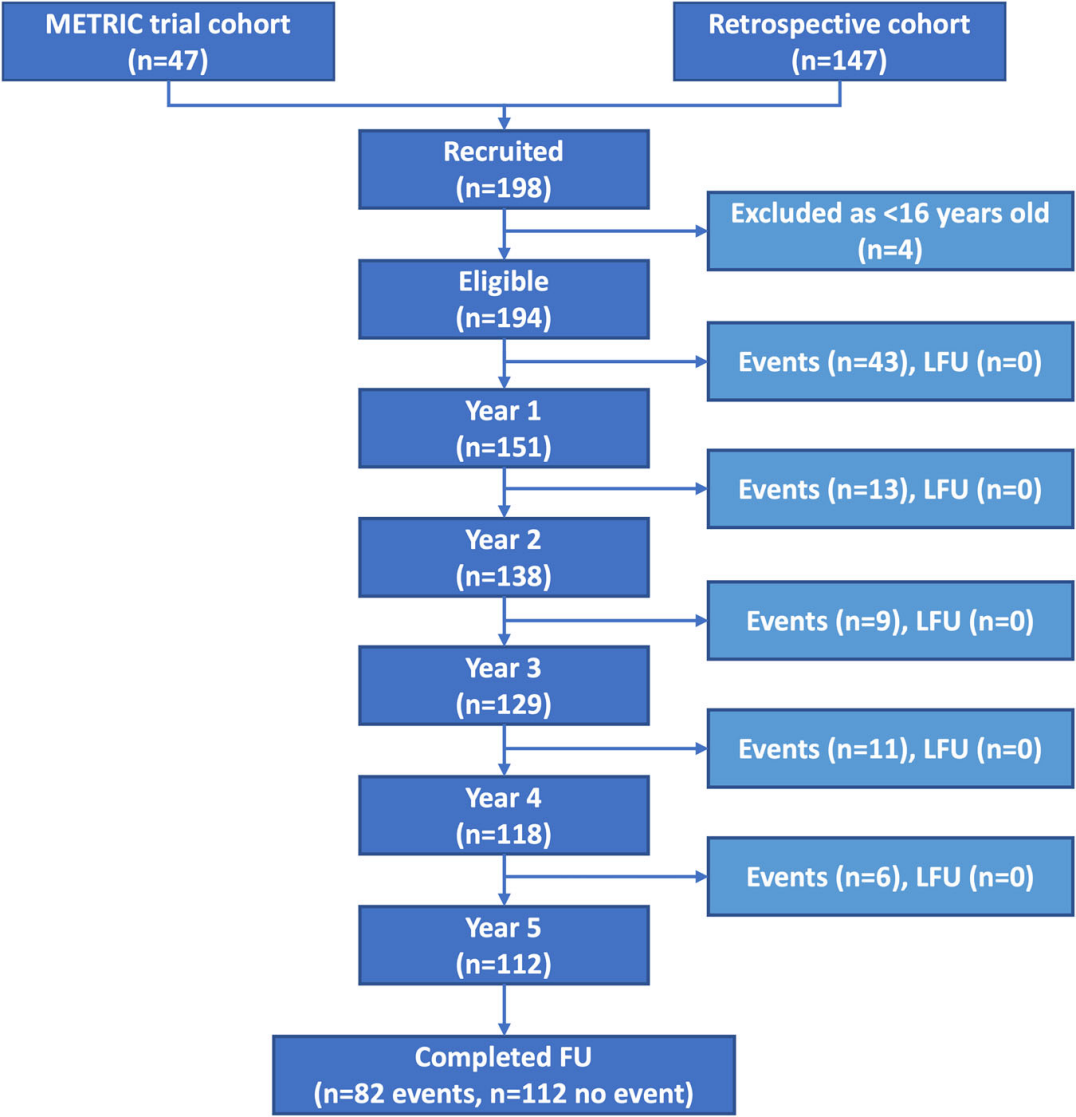
Participants flow through the trial, identifying participants by the timing of the first event during follow-up (> 90 days to 5 years). Event defined by MBDD. FU, follow up; LFU, lost to follow-up

The final cohort consisted of 194 patients; 93 (48%) were male and the median age was 29 (IQR 22 to 44 years) (Fig. 3 and Appendix 8). Overall, 42% (81/194) of participants developed MBDD between 90 days and 5 years post-diagnosis. An additional 25 patients were hospitalised within the first 90 days of diagnosis (and thus not considered MBDD for the purposes of prognostic modelling). Only 20% (39/194) developed disabling disease according to Liege criteria and 6% (12/194) developed B2 or B3 disease according to Montreal criteria (Appendix 9). The event rate using Liege and Montreal definitions of disabling disease was insufficient to perform predictive modelling; therefore, all data were analysed using the primary outcome measure (MBDD criteria).

Demographic and clinical characteristics of participants who developed MBDD within 5 years of diagnosis is shown in Appendix 10. Of a total 88 MBDD events, 43 (49%) occurred in year 1 and 6 (7%) occurred in year 5. Most MBDD events were hospitalisation due to a CD flare or complication (48 events; 55%) or due to use of corticosteroids (23 events; 26%) (Appendix 11).

### Clinical and MRE predictors of disabling disease

The number of patients developing MBDD according to the MRE scores is shown in Table 2.

**Table 2:** Number of participants who developed modified Beaugerie disabling disease (MBDD) within 5 years of diagnosis, stratified by prespecified MRE score predictors

Prespecified MRE score predictors	Did not develop MBDD	Developed MBDD	Total
	*N* = 113	*N* = 81	*N* = 194
Global MEGS	21 (8, 34)	24 (8, 37)	22 (8, 35)
Normalised global MEGS (%)	14 (5, 23)	17 (5, 25)	15 (5, 24)
Global sMARIA	5 (2, 8)	5 (2, 6)	5 (2, 7)
Normalised global sMARIA (%)	18 (7, 29)	18 (7, 21)	18 (7, 25)
Lémann index	2 (1, 4)	2 (1, 3)	2 (1, 3)
Normalised Lémann index (%)	10 (4, 21)	11 (6, 19)	11 (4, 20)

Data are median (IQR)

Scores were normalised to enable comparison of the scores on a standardised scale

*MBDD* modified Beaugerie disabling disease, *MRE* magnetic resonance enterography, *sMARIA* simplified magnetic resonance index of activity

### Years to events by predictors

Of those who developed MBDD, the median event-free (survival) time prior to this event was 0.82 (IQR 0.42, 2.75) years. The median event-free (survival) time to developing MBDD is shown in Appendices 12 and 13.

### Univariable Hazard ratio by clinical predictors

The univariable hazard ratio for clinical and MRI predictors of MBDD are summarised in Table 3. Only initial requirement for steroid therapy was associated with a higher risk of MBDD in both original and imputed data.

**Table 3:** Univariable hazard ratios of prespecified predictors for predicting the development of modified Beaugerie disabling disease (MBDD) within 5 years of diagnosis, using observed and imputed data

Prespecified predictors		Observed data			Imputed data		
		*N*	Hazard ratio (95% CI)	*p*-value	*N*	Hazard ratio (95% CI)	*p*-value
≥ 40 years of age		194	0.71 (0.42, 1.18)	0.185			
Female		194	1.01 (0.65, 1.57)	0.954			
Smoker		181	1.51 (0.94, 2.45)	0.092	194	1.52 (0.95, 2.44)	0.082
Weight loss ≥ 5 kg prior to diagnosis		171	0.89 (0.52, 1.52)	0.659	194	0.83 (0.48, 1.43)	0.509
Initial need for steroid therapy		194	2.11 (1.36, 3.26)	0.001			
Developed MBDD ≤ 90 days from diagnosis		194	1.38 (0.75, 2.54)	0.305			
Perianal disease		193	1.15 (0.61, 2.16)	0.674	194	1.15 (0.61, 2.18)	0.664
Severe endoscopic disease		181	1.00 (0.60, 1.66)	0.995	194	1.04 (0.62, 1.75)	0.869
Disease behaviour	B1	194	-	-			
	B2		1.38 (0.80, 2.40)	0.247			
	B3		1.39 (0.77, 2.51)	0.281			
Location of disease behaviour	Ileocolonic	194	-	-			
	Ileal/Upper tract		0.89 (0.55, 1.43)	0.619			
	Colonic		0.75 (0.39, 1.45)	0.392			
Normalised global MEGS (%)		194	1.01 (0.99, 1.02)	0.366			
Normalised global sMARIA (%)		194	1.00 (0.98, 1.01)	0.544			
Normalised Lémann index (%)		194	1.00 (0.99, 1.01)	0.888			
CRP level (mg/L)		166	1.00 (1.00, 1.01)	0.344	194	1.00 (1.00, 1.01)	0.348
WBC count (10^9^/L)		156	-	-	194	1.01 (0.94, 1.07)	0.877
Faecal calprotectin level (μg/g)		75	1.00 (1.00, 1.00)	0.892	194	1.00 (1.00, 1.00)	0.719
Haemoglobin level (g/L)		160	1.00 (0.98, 1.01)	0.674	194	1.00 (0.98, 1.01)	0.680
Platelet count (10^9^/L)		151	-	-	194	1.00 (1.00, 1.00)	0.762

Scores were normalised to enable comparison of the scores on a standardised scale. Min-max normalisation rescales values between 0% and 100%. This ensures the lowest score becomes 0% and the highest becomes 100%, keeping relative differences intact. It was not possible to fit a univariable model for platelet count or WBC count because convergence in the model could not be achieved

*CRP*  C-reactive protein, *MEGS* Magnetic Resonance Enterography Global Score, *sMARIA* Simplified Magnetic Resonance Index of Activity, *WBC* white blood cell

**Fig. 4:**
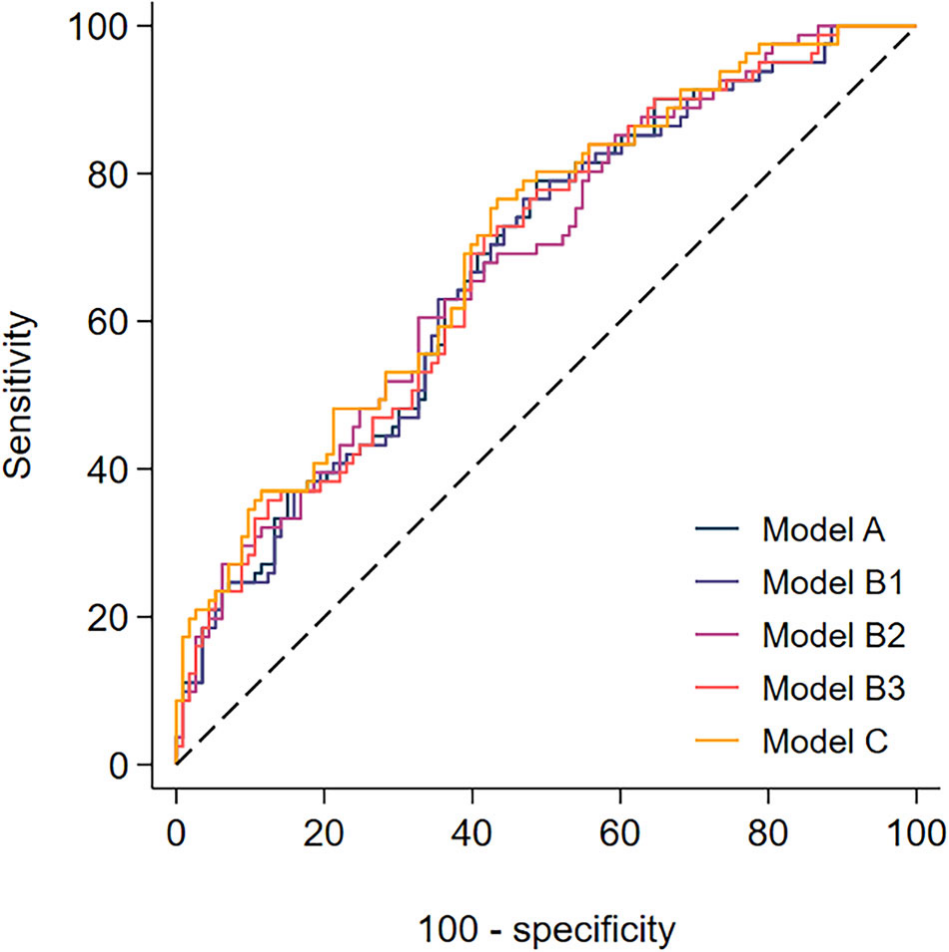
ROC plot and area under the curve (AUC) of prognostic models for predicting the development of MBDD within 5 years of diagnosis

The ROC curve for the multivariable models (models A (clinical predictors), B1 (clinical predictors plus MEGs), B2 (clinical predictors plus sMARIA), B3 (clinical predictors plus Lemann) and C (clinical predictors plus blood and stool predictors)) are shown in Fig. 4 and Appendices 14 to 20. Sensitivity and specificity of the models were similar across all risk thresholds.

### MBDD event-free Kaplan–Meier according to prognostic models

The predictive performance of all multivariable models was assessed using the two a priori defined clinical risk groups, and are shown in Appendix 21.

Table 4 and Appendix 14 show the sensitivity and specificity of each model for identifying patients in each of the two predefined risk groups. Overall, there was no statistically significant difference in sensitivity between model A (clinical predictors) and each of the other models, for either patient risk group (Appendix 12). For patient risk group 2, models B1 (clinical predictors plus MEGs), B3 (clinical predictors plus Lemann) and C (clinical predictors plus blood and stool predictors) had significantly lower specificity than the baseline model A (clinical predictors) (Appendix 14). Overall, prediction did not improve when either MRE scores (MEGS, sMARIA, LI), or blood or stool parameters were added to the baseline model A.

**Table 4:** Sensitivity and specificity of prognostic models, stratified by risk group definition for the development of modified Beaugerie disabling disease (MBDD)

Prognostic model	Risk group definition	Risk group	Percentage did not develop MBDD (95% CI)	Did not develop MBDD	Developed MBDD	Sensitivity (95% CI)	Specificity (95% CI)
				*N* = 113	*N* = 81		
A	1	Low	65 (55, 73)	75	41	49 (39, 60)	66 (57, 74)
		High	49 (38, 60)	38	40		
	2	Low	79 (65, 88)	40	11	86 (77, 92)	35 (27, 45)
		High	51 (43, 59)	73	70		
B1	1	Low	66 (56, 74)	76	40	51 (40, 61)	67 (58, 75)
		High	48 (36, 58)	37	41		
	2	Low	83 (67, 91)	33	7	91 (83, 96)	29 (22, 38)
		High	52 (44, 60)	80	74		
B2	1	Low	67 (57, 74)	77	39	52 (41, 62)	68 (59, 76)
		High	47 (35, 57)	36	42		
	2	Low	82 (66, 91)	31	7	91 (83, 96)	27 (20, 36)
		High	53 (45, 60)	82	74		
B3	1	Low	66 (57, 74)	77	40	51 (40, 61)	68 (59, 76)
		High	47 (36, 58)	36	41		
	2	Low	83 (67, 91)	33	7	91 (83, 96)	29 (22, 38)
		High	52 (44, 60)	80	74		
C	1	Low	68 (58, 75)	79	38	53 (42, 64)	70 (61, 78)
		High	45 (33, 55)	34	43		
	2	Low	84 (69, 92)	36	7	91 (83, 96)	32 (24, 41)
		High	51 (43, 59)	77	74		

For risk group definition 1, the high-risk group included the top 40% of participants with the greatest predicted risk from the model. For risk group definition 2, the high-risk group included participants with an absolute risk greater than or equal to 10%

### Model performance in a hypothetical 1000 participants

Performance of the various models was assessed in a hypothetical cohort of 1000 patients for risk group definitions 1 and 2, and are shown in Appendix 22. For example, model A (clinical predictors) would predict that 402 patients develop MBDD within 5 years of diagnosis, of whom 217 would be correct predictions and 185 incorrect false positives.

### Principal components analysis (PCA) analysis

Of 11 MRI features included in the PCA (Appendix 7), four components accounted for more than 70% of the total variance. A loading table with the full list of MRE features is shown in Appendix 7. Including these four PCs in model A (clinical predictors) resulted in collinearity with clinical variables, preventing model development due to a lack of model convergence.

### Exploratory analyses

Smoking at diagnosis was significantly positively associated with future bowel resection (OR 2.38 (95% CI 1.14, 4.98)) within 5 years (Appendix 23). A possible association between maximum segmental MEGS (OR 5.36 (95% CI 2.34, 12.29)) with subsequent bowel resection would require evaluation in future datasets due to the large width of the 95% CI (Appendix 23). Two other estimates for prediction of bowel resection are not interpretable in this study despite apparent statistically significant associations, as indicated by 95% CI including values above 20 HR (maximum segmental sMARIA, and presence of Montreal B2/B3 disease at diagnosis). We found no evidence that starting biologic therapy within 180 days of diagnosis protected against MBDD, irrespective of segmental or global sMARIA score (Appendix 24).

## Discussion

A priori, we hypothesised that either active inflammation or established bowel damage on baseline MRE, as measured by sMARIA, MEGS or the LI, would help prognosticate disabling CD when incorporated into a multivariable model comprising standard, commonly collected clinical predictors. However, we found that no MRE scoring system improved the standard baseline model. Furthermore, in univariable analysis, an initial requirement for steroid therapy was the only clinical predictor of MBDD that achieved statistical significance.

Our findings imply that neither active inflammation nor bowel damage, when measured by sMARIA, MEGS, or LI, predicts MBDD. The sMARIA quantifies CD activity, disease severity, and treatment response, and has been externally validated against a range of reference standards [34–36]. It has good performance characteristics compared with endoscopy, although specificity is lower when compared to histological reference standards [36]. MEGS comprises both imaging markers of active inflammatory disease and established bowel damage, and has also been tested extensively against multiple reference standards [37–40]. The LI differs from both sMARIA and MEGS by providing an assessment of structural bowel damage, with lesser weighting placed on the severity of disease activity and mucosal inflammation [41]. MRE also identifies abnormalities that persist in intestinal segments even after endoscopic remission of CD has occurred, implying that intestinal damage is established [42]. Such MRE findings include persistent mural thickening, mural fat deposition, creeping fat, and strictures. The relevance of these findings, especially in the context of future disease outcomes, remains unclear.

That we did not find an association between MRE features and subsequent disabling disease may potentially reflect inherent limitations of our definition of disabling disease, rather than a lack of predictive capability. There is no universally accepted disease severity classification or validated definition for severe or disabling CD. Therefore, we were obliged to employ a range of definitions of disabling disease. We adopted a modified version of the relatively inclusive Beaugerie description [6], removing the commencement of biologic therapy. We took this decision a priori after careful consideration, our rationale being that this represented a ‘top-down’ approach that will likely become increasingly adopted as standard of care following the PROFILE trial [9]. Thus, biologic therapy does not necessarily indicate severe disease, but rather a desire to prevent it. We considered the possibility that patients who were treated aggressively with biologics and immunomodulators at diagnosis may diminish the proportion ultimately progressing to MBDD, but we found no evidence to support this, although our study was not designed to test this. Furthermore, many of the Beaugerie events, largely hospitalisation, occurred within 90 days, and are likely related to the initial diagnostic episode and disease control. Because patients with severe disease at diagnosis cannot benefit from a model developed to predict future severe disease, we excluded this group. The purpose of our model was to facilitate individual patient management as top-down treatment increasingly becomes the standard of care [9]. Another challenge in prognosticating CD is the dichotomy that often exists between symptoms and detectable intestinal disease activity [43–45]. Given the lifelong relapsing and remitting nature of CD, it is likely that many patients adapt to tolerate the condition and may be unwilling to divulge symptomatology in fear of undergoing resective bowel surgery [46]. Furthermore, if imaging findings suggest that surgery is required but the patient is largely asymptomatic, they may well decline intervention. Two of the four main categories for defining disabling disease that we used were based on symptoms and the need for surgery, so this may explain why we did not find imaging metrics to be predictive. Indeed, perhaps imaging ought to be an endpoint for disabling disease, as it is a more objective and reproducible measure. Further work should investigate the ability of imaging specifically to predict stricturing or penetrating disease as outcomes independent of symptoms or surgical intervention.

Other studies have considered a potential prognostic role for MRE in CD, although not specifically in newly diagnosed patients. Most have used intestinal surgery to indicate adverse outcomes, which is potentially flawed since planned surgery is often highly efficacious, especially for limited CD [47]. Fiorino et al studied 142 patients in a dual-centre prospective study [20]. Using univariate analysis, they found that bowel damage (defined as intestinal strictures, fistulae or abscesses) was associated with significantly higher risk of hospitalisation and surgery during a median follow-up period of just under 5 years. Similarly, the LI was an independent predictor for disease progression and the need for subsequent surgery. Patients were eligible for recruitment if MRE was performed within 2 years of diagnosis, which differs from our study since we imaged at initial diagnosis. A single-centre study of 112 patients with relapsed CD, on univariate analysis, found that established bowel damage on MRE was associated with future surgical resection [21]. Another single centre study of 52 patients (that did not distinguish between new and established diagnoses), reported that restricted diffusion, increased upstream dilatation from a stricture, complex fistula, perienteric inflammation, fibrofatty proliferation, and increased length of disease involvement on MRE, were significantly more common in patients having surgery subsequently [23]. However, it is unclear if these findings were also statistically significant in univariate analysis or if this was a misinterpretation of individual variable coefficients from a multivariable model [48]. Most recently, in a post hoc analysis, Fernández-Clotet et al followed 89 patients for 2 years post-biologic initiation, who underwent MRE at 46 weeks [49]. Severe inflammatory lesions in any segment, stenosis and/or abscesses and fistulas, as well as creeping fat on MRE, were associated with poor clinical outcomes. However, MRE at diagnosis was unavailable because patients were not newly diagnosed.

While we found MRE had no predictive potential for modified Beaugerie criteria, in an exploratory analysis, we did find some evidence (as might be expected) that patients with Montreal classification B2/3 at diagnosis (i.e., stricturing or penetrating disease) were more likely to need subsequent bowel resection. We also found an association between higher maximal segmental MRE disease MEGS scores at diagnosis and subsequent surgery, although confidence intervals were wide and larger studies powered around this analysis are needed to confirm this finding. Of note, because we investigated newly diagnosed patients, by definition, they are early in their disease trajectory [21]. To the best of our knowledge, this is the first study to investigate MRI as a predictive biomarker of disabling CD, which exclusively investigated newly diagnosed patients without severe disease at baseline, and tested MRE against commonly collected clinical predictors. Generalisability was enhanced by a large cohort recruited from multiple hospitals, with multiple radiologists scoring MRE examinations, thereby closely reflecting routine clinical practice.

There are also noteworthy limitations. A priori, we had intended to employ the more stringent Liège [28] and Montreal behaviour criteria as endpoints for severe/disabling disease [29]. However, the event rate was too low to develop predictive models. The lower-than-expected Beaugerie event rate (42% vs our anticipated 50% to 60%) may reflect greater upfront use of biologics, although we stress our exploratory analyses found no specific evidence for this. We recognise that predicting outcomes based on imaging is likely to be compromised because imaging directs treatment; identification of severe disease leads to more effective treatment and better outcomes. The COVID-19 pandemic obliged us to reduce the original METRIC target recruitment from 167 to 131, necessitating a corresponding increase in the retrospective cohort to 76 patients. Another limitation of this trial is that we did not assess inter-observer agreement for the various activity scores, and it is plausible that there was variation between the 11 readers. At the time of the protocol development, there were strong data supporting high inter-observer agreement for MRE [50–52], but recent work has challenged the reproducibility of MRE findings [53]. Future work is needed to clarify this.

## Conclusions

Our work suggests that current MRE activity and damage scores at diagnosis cannot reliably predict whether patients will subsequently develop disabling CD. Notwithstanding this finding, MRE remains an essential tool for diagnosis and monitoring.

## Supplementary information

Supplementary information

## Abbreviations

CD: Crohn’s disease
LI: Lémann index
MBDD: Modified Beaugerie disabling CD
MEGS: Magnetic Resonance Enterography Global Score
MRE: Magnetic resonance enterography
PCA: Principal component analysis
sMARIA: Simplified MR Index of Activity
NHS: UK National Health Service
