# Supplementary material for: Magnetic resonance enterography to predict subsequent disabling Crohn’s disease in newly diagnosed patients (METRIC-EF)—multivariable prediction model, multicentre diagnostic inception cohort
Source: Eur Radiol. 2025 May 14;35(11):7333–45. doi: 10.1007/s00330-025-11636-8 (PMC12559088; doi:10.1007/s00330-025-11636-8)
Supplement: Supplementary file 1 — Supplementary information [file 330_2025_11636_MOESM1_ESM.pdf]

Magnetic resonance enterography to predict subsequent disabling Crohn's disease in newly diagnosed patients (METRIC-EF) – multivariable prediction model, multicentre diagnostic inception cohort

ELECTRONIC SUPPLEMENTARY MATERIAL

Supplemental file to Magnetic resonance enterography to predict disabling disease in newly diagnosed Crohn's disease (METRIC-EF) – multivariable prediction model, multicentre diagnostic inception cohort

Appendix 1

Eligibility criteria for the trial.

METRIC cohort: Inclusion criteria

All confirmed new diagnoses from METRIC were eligible for the present study; inclusion criteria were therefore equivalent to those of METRIC:

- Patients aged 16 years or more.
- new CD diagnoses (within 3 months of time of recruitment to METRIC), based on standard endoscopic, histological, clinical, and radiological findings.

Additional retrospective cohort: Inclusion criteria

We added a retrospective cohort to the METRIC accruals to achieve the required sample size.

Inclusion criteria for the retrospective cohort were:

- Patients 16 year or more with newly diagnosed CD, based on endoscopic, histological, clinical and radiological findings
- MRE acquired according to METRIC standard minimum sequence dataset, and performed either <3 months before or after diagnosis
- Normal institutional practice is to perform MRE in all new diagnoses of CD.
- At least 4 years clinical follow-up data available

Sites who were not part of the original METRIC trial were eligible to be recruitment sites for the retrospective cohort if they fulfilled all eligibility criteria.

Exclusion criteria

Exclusion criteria for METRIC (and so carried forward) were:

- Any psychiatric or other disorder likely to impact on informed consent
- Evidence of severe (non-Crohn's) co-morbidities which makes it undesirable for the patient to participate in the study
- Pregnancy
- Contraindication to MRI (e.g., cardiac pacemaker, severe claustrophobia, inability to lie flat)
- Final diagnosis other than CD
- Enrolled in the METRIC study but not part of the final new diagnosis cohort

Appendix 2

Required and optional sequences for the magnetic resonance enterography studies.

*Optional for retrospective cohort. DWI = diffusion weighted imaging, GRE = gradient echo, FSE = fast spin echo

This Table has been reproduced with from Kumar et al.[1] This is an Open Access article distributed in accordance with the terms of the Creative Commons Attribution (CC BY 4.0) license, which permits others to distribute, remix, adapt and build upon this work, for commercial use, provided the original work is properly cited. See: http://creativecommons.org/licenses/by/4.0/. This includes minor additions and formatting changes to the original table/Figure.

Required	Optional
Coronal balanced steady-state GRE	Axial balanced steady-state GRE
Axial echo-planar FSE	Dynamic steady-state free precession GRE motility
Coronal echo-planar FSE	
Coronal echo-planar FSE with fat suppression	Axial radio-frequency-spoiled 3D GRE with fat suppression
Axial DWI (b50 and b600)*	Additional b values
Coronal pre- and post-gadolinium radio-frequency-spoiled 3D GRE (60-70 seconds)*	Axial post-gadolinium radio-frequency-spoiled 3D GRE

Appendix 3

Calculation of the magnetic resonance enterography score (MEGS), simplified magnetic resonance index of activity (sMARIA), and Lémann index (LI).

This Table has been reproduced with from Kumar et al.[1] This is an Open Access article distributed in accordance with the terms of the Creative Commons Attribution (CC BY 4.0) license, which permits others to distribute, remix, adapt and build upon this work, for commercial use, provided the original work is properly cited. See: http://creativecommons.org/licenses/by/4.0/. This includes minor additions and formatting changes to the original table/Figure.

Mural features	0	1	2	3	Score
Mural thickness	<3mm	>3-5mm	>5-7mm	>7mm	a
Mural T2 signal (oedema)	Normal	Minor increase	Moderate increase	Large increase	b
Perimural T2 signal	Normal	Increased signal but no fluid	Small (≤2mm) fluid rim	Large (>2mm fluid rim)	c
Contrast enhancement: amount	Normal	Minor increase	Moderate increase	Large increase	d
Contrast enhancement: pattern	N/A or homogenous	Mucosal	Layered		e
Haustral loss (colon only)	None	<1/3 segment	1/3 to 2/3 segment	>2/3 segment	f
				**Mural score for that segment**	**a+b+c+d+e+f = g**
**Multiplication factor**	**1**	**1.5**	**2**	**TOTAL SEGMENTAL SCORE g * multiplication factor**	
Length of disease in that segment	<5cm	5-15cm	>15cm		

*Each enteric segment (jejunum; proximal ileum; terminal ileum; caecum; ascending colon; transverse colon; descending colon; sigmoid colon; rectum) is scored separately. The segmental score is then multiplied by a factor depending on the length of disease involvement in that segment. Finally, scores for extramural features are added, giving a total score (maximum possible = 296). Sum all segments, then add extramural score on a per-scan basis; 5 points for each of: (1) lymph nodes >1cm short axis, (2) comb sign (linear structures on the mesenteric border of an affected bowel segment), (3) abscess and (4) fistula.

Feature	Description
Mural thickness	Binary: Measured in mm using software calipers, scored as abnormal if >3mm
Mural oedema	Binary: present if there is high signal intensity on T2 sequences with fat saturation, compared with normal-appearing loops
Fat stranding	Binary: present if there is loss of the normal sharp interface between the intestinal wall and mesentery, with oedema/fluid in the perienteric fat
Ulceration	Binary: present if mucosal surface has a deep depression, visible on 2 MRI sequences
**sMARIA score for that segment**	**= 1 point for each of mural thickness, mural oedema, and fat stranding; 2 points for ulceration (maximum 5 points per segment)**

**Surgical interventions†**						
**Organ**	**Method of assessment**	**n***	**Segment**	**Grade 1**	**Grade 2**	**Grade 3**
Upper tract	History	3	Oesophagus, stomach, duodenum	-	Bypass diversion or strictureplasty	Resection
Small bowel	History	20	Each 20cm SB segment	-	Bypass diversion or strictureplasty	Resection
Colon / rectum	History	6	Each colonic segment	-	Stoma, bypass diversion or strictureplasty	Resection

† This information was collated from patient records, although a relevant past surgical history was very rare since included patients were, by definition, those with a new diagnosis of Crohn's disease. Prestenotic dilatation defined if > 3 cm.

**n = number of segments within a particular organ*

Stricturing lesions						
**Organ**	**Method of assessment**	**n**	**Segment**	**Grade 1**	**Grade 2**	**Grade 3**
Upper tract	MRI	2	Stomach, duodenum	Wall <3mm; segmental enhancement without prestenotic dilatation	Wall thickening ≥3mm or mural stratification with no prestenotic dilatation	Stricture with prestenotic dilatation
Small bowel	MRI	20	Each 20cm SB segment	Wall <3mm; segmental enhancement without prestenotic dilatation	Wall thickening ≥3mm or mural stratification with no prestenotic dilatation	Stricture with prestenotic dilatation
Colon / rectum	MRI	6	Each colonic segment	Wall <3mm; segmental enhancement without prestenotic dilatation	Wall thickening ≥3mm or mural stratification with no prestenotic dilatation	Stricture with prestenotic dilatation or >50% of the lumen

Penetrating lesions						
**Organ**	**Method of assessment**	**n**	**Segment**	**Grade 1**	**Grade 2**	**Grade 3**
Upper tract	MRI	2	Stomach, duodenum	-	Deep transmural ulceration	Phlegmon or fistula
Small bowel	MRI	20	Each 20cm SB segment	-	Deep transmural ulceration	Phlegmon or fistula
Colon / rectum	MRI	6	Each colonic segment	-	Transmural ulceration	Phlegmon or fistula

Appendix 4

Alternative definitions of disabling disease

The Liège criteria were met if any of the following occurred:

- Development of complex perianal disease.
- Any colonic resection.
- Two or more small bowel resections.
- A single small bowel resection of >50cm.
- Definitive stoma.

Complex perianal disease was defined as per the American Gastroenterological Association. Sandborn WJ, Fazio VW, Feagan BG, et al. AGA technical review on perianal Crohn's disease. *Gastroenterology*. 2003;125(5):1508-1530.

The Montreal behaviour criteria classify CD as either inflammatory (B1), stricturing (B2) or penetrating (B3). Stricturing disease was defined as a fixed luminal narrowing of >50% relative to normal proximal bowel. Penetrating disease was defined as an intra-abdominal or enterocutaneous fistula, inflammatory mass, or abscess.

Appendix 5 Potential clinical predictors at diagnosis.

- Age
- Smoking status
- Sex
- Disease behaviour (stricturing or penetrating)
- Perianal disease
- Severe endoscopic disease (defined as deep ulcerations covering more than 10% of the mucosal area of at least one intestinal segment)
- Location of disease (ileal, colonic, ileocolonic, upper tract)
- Initial need for steroid therapy
- Weight loss of at least 5kg prior to diagnosis
- CRP
- White blood cell (WBC) count
- Faecal calprotectin
- Haemoglobin
- Platelet count
- Development MBDD ≤90 days from diagnosis

Appendix 6

Sample size and justification

Assumptions

We assumed that the prevalence of MBDD was approximately 55 to 60%; this was informed primarily by the external validation cohort of the Beaugerie descriptors, in which 57% of 361 participants had developed disabling disease within 5 years of diagnosis.[1] In support, a local audit of 33 newly diagnosed patients at one METRIC recruitment centre at the trial planning stage found 5 of 33 (15%) patients met the definition by mean 11.3 months, giving 16% at 1 year. Extrapolation to 5 years gave 58% prevalence, similar to that expected from the literature.[1] The sample size was based on including 207 participants newly diagnosed with CD; 207 participants provided 114 to 124 patients developed MBDD; the smaller proportion defines the minimum sample size for powering a modelling study. During the study, due to problems obtaining consent for additional follow up due to the COVID-19 pandemic, the Trial Management group reduced the original target recruitment from 167 to 131 in the prospective METRIC cohort, with a corresponding increased target of seventy-six participants from the retrospective cohort. We anticipated that this sample size would provide between 114 and 124 patients developing MBDD. We would increase the number of the retrospective cohort to meet the 207-participant target if recruitment to the METRIC cohort was below 131.

Adequacy of this number of events/non-events

Calculating sample sizes for prognostic studies suffers from a relative lack of readily applied methods suitable for all study designs, since sample size for development depends on whether the primary aim is to select potential variables for a new model (via univariable significance within a dataset), or to evaluate a model where the variables have been pre-specified and are therefore fixed. In the present study, we fixed predictors since we were explicit that we would evaluate 3 MRE severity scores in the context of a model using fixed clinical predictors. Therefore, recommendations for sample sizes relevant to external validation were most appropriate. Accordingly, the literature suggested that we required 80 to 100 events for model evaluation where predictors were pre-specified and fixed.[2] This also provided sufficient power to assess whether addition of the 3 MRE severity scores enhanced prediction, under the hitherto widely-used "rule-of-thumb" of 10 to 20 events per predictor.[3] We are aware of recent methods to calculation model development and external validation sample size, but these were not reported in 2017, when the present study was powered.[4]

Power for secondary outcomes

Other definitions of adverse outcome

Development of Montreal severe disease was estimated to be 43% at 5 years.[5]

Development of Liège disabling disease was estimated to be 20% at 5 years.[1] This provided approximately 41 events for the present study which was likely insufficient to develop meaningful prognostic models. Accordingly, we planned that analysis for this endpoint would be descriptive only, unless our assumptions proved incorrect and sufficient events satisfying this definition had been accumulated.

Identification of the most important MRE variables for model inclusion

We used principal component analysis (PCA) to reduce the number of individual MRE features to ideally two or three eigenvector variables, for subsequent addition to the clinical predictor only model. This allowed us to determine how adding MRE features affected model performance.

Retention

Participants did not undergo additional testing to enter this study. Only data obtained during routine clinical care were necessary to both define disabling disease and provide variables for model inclusion. Where participants were lost to local follow up, participants' GP were contacted in an attempt to obtain routine clinical information, post consent (this was only applicable to the METRIC cohort and those patients on retrospective cohort who had provided consent).

Statistical Methods – Outcomes

Primary outcome

Comparative predictive ability of prognostic models incorporating MRE scores (MEGS, sMaRIA and Lémann index) versus a model based on clinical predictors alone for the development of MBDD within 5 years of diagnosis.

We developed a Royston-Parmer flexible parametric multivariable prognostic model using the following pre-specified clinical predictors (based on a prior literature review and in consultation with the trial investigators):

- age at diagnosis (<40, ≥40 years)
- smoking history
- sex
- disease status at diagnosis (stricturing disease, perianal disease, severe endoscopic disease)
- location of disease (Ileal, colonic, ileocolonic, upper GI tract disease)
- initial need for steroid therapy
- weight loss of at least 5kg prior to diagnosis
- C-reactive protein
- white blood cell count,
- faecal calprotectin
- haemoglobin
- platelet count

There were five prespecified continuous predictors, including CRP level, WBC count, faecal calprotectin level, haemoglobin level, and platelet count. We determined whether we should include the predictors as linear or to use fractional polynomials. Due to high levels of missing WBC count and faecal calprotecin levels, we could not investigate if fractional polynomial was appropriate, so assumed a linear relationship. For the remaining predictors, we calculated the best fractional polynomial models by searching through all power combinations. Then, we calculated p-values by comparing the deviance of the linear and FP model 1 against the deviance of FP model 2 (lowest deviance). We determined that retaining linear continuous predictors was the most efficient.

We retained categorical predictors as in the clinical report form, except for when modelling required us to combine specific levels of a predictor. For location of disease behaviour, we combined the ileal and upper tract levels due to small number of patients (N=4) with disease in the upper tract.

Seventy-five percent of participants had ≥1 predictor value missing. Because missing values were likely to be "missing at random" based on other participant variables, and to avoid loss in efficiency, we imputed values for smoking status, weight loss ≥5 kg prior to diagnosis, perianal disease, severe endoscopic disease, CRP level, WBC count, faecal calprotectin, haemoglobin level, and platelet count using multiple imputation by chained equations (mi impute command in Stata 18).[6] We created 20 imputed datasets from a set of imputation models constructed from all predictors and outcomes (event indicator and Nelson-Aalen estimator for time to event).

We based an improvement in model performance on an increase in the number of patients correctly predicted to develop MBDD, relative the clinical predictor only model. We used sensitivity, specificity, and net benefit as measures of model performance. We conducted internal validation using 200 bootstrap samples (sampling with replacement) or until estimates remained stable. We describe the results from internal validation below. We did not adjust for optimism. Statistical significance was based on Wilson's 95% CI. We calculated p-values for differences in sensitivity and specificity using McNemar's test.

Internal validation of prognostic models

Prognostic model	Data	Mean linear predictor (SD)	Harrell's C-statistic	R2 (95% CI)	D-statistic	C-slope (95% CI)	Heuristic shrinkage factor
B	Observed	0.30 (0.61)	0.66	0.19 (0.07, 0.33)	1.00	1.00 (0.56, 1.44)	0.977
	Imputed	0.12 (0.48)	0.67	0.14 (0.05, 0.25)	0.83	1.04 (0.71, 1.37)	0.980
A1	Observed	0.32 (0.61)	0.66	0.19 (0.07, 0.33)	1.00	1.00 (0.56, 1.44)	0.976
	Imputed	0.13 (0.48)	0.67	0.14 (0.04, 0.23)	0.83	1.04 (0.81, 1.27)	0.979
A2	Observed	0.13 (0.63)	0.67	0.22 (0.09, 0.36)	1.08	1.00 (0.57, 1.43)	0.976
	Imputed	-0.06 (0.51)	0.68	0.15 (0.06, 0.26)	0.86	1.04 (0.72, 1.35)	0.979
A3	Observed	0.24 (0.61)	0.66	0.20 (0.08, 0.34)	1.02	1.00 (0.56, 1.44)	0.976
	Imputed	0.09 (0.48)	0.67	0.14 (0.05, 0.24)	0.83	1.04 (0.77, 1.31)	0.979
B1	Observed	5.72 (1.22)	0.76	0.47 (0.22, 0.64)	1.91	1.00 (0.57, 1.43)	0.965
	Imputed	-0.24 (0.50)	0.68	0.16 (0.06, 0.28	0.90	1.18 (0.86, 1.50)	0.974

Secondary outcomes

<u>Secondary outcome 1</u>

Comparative predictive ability of prognostic models including MRE scores (MEGS, sMARIA, and LI) versus a model based on clinical predictors alone to predict the development of disabling CD within 5 years of diagnosis, defined by Montreal behaviour and Liège criteria. We conducted modelling using the same methods as in the primary outcome. Models were only developed if the number of patients developing disabling CD was adequate. Otherwise, we provided descriptive statistics.

Secondary outcome 2

Identification of the best combination of individual MRE features for predicting disabling CD (all definitions) within 5 years of new diagnosis. PCA was used to combine multiple MRE parameters into a small number of Eigenscores variables. This allowed a larger number of features to be combined without compromising statistical power. The most influential imaging features were identified for further simplification of MRE variables included in modelling. Methods were as in the primary outcome, and the statistical significance of including MRE features were evaluated based on improvement of model fit (BIC) in comparison to the standard model, with additional model performance reported as appropriate.

Model testing

To provide additional clinical relevance for potential model implementation, we formed a group from the trial group, including 3 gastroenterologists and 2 radiologists. The group in consensus defined a priori how the models could be best utilised in clinical practice. Specifically, following guidance from the study statisticians, they set two risk group definitions for identifying patients at high- and low-risk of developing disabling disease which they felt would have clinical utility.

For risk group definition 1, the high-risk group included the top 40% of participants with the greatest predicted risk from the model. For risk group definition 2, the high-risk group included participants with an absolute risk greater than or equal to 10%. The absolute risk threshold is determined by sorting the participants by predicted risk, and then using the predicted risk of the 8th (10% of 81) participant who developed MBDD as the threshold.

Appendix 7

Variable loadings for principal components of prespecified predictors.

Prespecified predictors	Component 1	Component 2	Component 3	Component 4
Maximum mural thickness (MEGS)	0.36	-0.24	0.1	-0.13
Maximum mural T2 signal (oedema) (MEGS)	0.4	-0.14	0.05	-0.03
Maximum contrast enhancement pattern	0.31	-0.15	0.07	0.38
Maximum length of disease (MEGS)	0.38	-0.22	-0.01	-0.12
Abscess (MEGS)	0.25	0.56	0.19	0.09
Maximum fat stranding (sMARIA)	0.36	-0.13	0.1	0
Number of abnormal segments	0.33	-0.2	-0.11	-0.39
Maximum upper tract and small bowel stricturing	0.2	-0.05	-0.2	0.67
Maximum colon stricturing	0.03	0.12	0.89	-0.03
Maximum upper tract and small bowel penetrating	0.29	0.52	-0.15	0.18
Maximum colon penetrating	0.22	0.44	-0.27	-0.43

Appendix 8 Demographic and clinical characteristics of participants who developed modified Beaugerie disabling disease (MBDD) within 5 years of diagnosis.

**Demographic and clinical characteristics**		**Did not develop MBDD N=113**	**Developed MBDD N=81**	**Total N=194**
Age (years)		31 (22, 49)	27 (22, 37)	29 (22, 44)
Sex	Male	54 (48)	39 (48)	93 (48)
	Female	59 (52)	42 (52)	101 (52)
Medication administered within 5 years from diagnosis*	Aminosalicylate	52	37	89
	Biologic	80	113	193
	Immunomodulator	143	117	260
	Other	11	27	38
	Steroid	104	156	260

*Participants could be administered more than one of the same medications within 5 years from diagnosis

MBDD = modified Beaugerie disabling disease

Data are n (%) or median (IQR)

Appendix 9

Number of participants who developed disabling disease within 5 years of diagnosis, according to the modified Beaugerie, Montreal B2 or B3, and Liège criteria.

**Years from diagnosis to developing disabling disease**	**Modified Beaugerie criteria (%)**	**Montreal B2 or B3 (%)**	**Liège criteria (%)**
	**n=81**	**n=12**	**n=39**
1	43 (52)	2 (17)	28 (72)
2	13 (16)	1 (8)	3 (8)
3	9 (11)	5 (42)	4 (10)
4	11 (14)	3 (25)	3 (8)
5	6 (7)	1 (8)	1 (3)

Appendix 10

Demographic and clinical characteristics of participants who developed modified Beaugerie disabling disease (MBDD) within 5 years of diagnosis stratified by prespecified clinical predictors. Data are n (%) or median (IQR). CRP = C-reactive protein, MBDD = modified Beaugerie disabling disease, WBC = white blood cell.

**Prespecified clinical predictors**		**Did not develop MBDD**	**Developed MBDD**	**Total**
		n=113	n=81	n=194
Age category (years)	<40	76 (67)	62 (77)	138 (71)
	≥40	37 (33)	19 (23)	56 (29)
Sex	Male	54 (48)	39 (48)	93 (48)
	Female	59 (52)	42 (52)	101 (52)
Smoking status	Non-smoker	81 (72)	49 (60)	130 (67)
	Smoker	22 (19)	25 (31)	47 (24)
	Missing	10 (9)	7 (9)	17 (9)
Weight loss ≥5 kg prior to diagnosis	Absent	71 (63)	51 (63)	122 (63)
	Present	28 (25)	18 (22)	46 (24)
	Missing	14 (12)	12 (15)	26 (13)
Initial need for steroid therapy	Absent	84 (74)	43 (53)	127 (65)
	Present	29 (26)	38 (47)	67 (35)
Developed MBDD ≤90 days from diagnosis	Absent	100 (88)	69 (85)	169 (87)
	Present	13 (12)	12 (15)	25 (13)
Perianal disease	Absent	100 (88)	70 (86)	170 (88)
	Present	12 (11)	11 (14)	23 (12)
	Missing	1 (1)	0 (0)	1 (0)
Severe endoscopic disease	Absent	74 (65)	56 (69)	130 (67)
	Present	27 (24)	20 (25)	47 (24)
	Missing	12 (11)	5 (6)	17 (9)
Disease behaviour	B1	80 (71)	50 (62)	130 (67)
	B2	17 (15)	17 (21)	34 (18)

	B3	16 (14)	14 (17)	30 (15)
Location of disease	Ileocolonic	52 (46)	42 (52)	94 (48)
	Ileal/Upper tract	41 (36)	28 (35)	69 (36)
	Colonic	20 (18)	11 (14)	31 (16)
CRP level (mg/L)	n (%)	87 (77)	75 (93)	162 (84)
	Median (IQR)	12 (4, 39)	16 (6, 56)	14 (6, 46)
WBC count (10^9/L)	n (%)	82 (73)	70 (86)	152 (78)
	Median (IQR)	9 (8, 12)	9 (7, 12)	9 (8, 12)
Faecal calprotectin level (μg/g)	n (%)	43 (38)	30 (37)	73 (38)
	Median (IQR)	527 (108, 600)	521 (196, 600)	527 (132, 600)
Haemoglobin level (g/L)	n (%)	86 (76)	70 (86)	156 (80)
	Mean (SD)	126 (18)	125 (18)	126 (18)
Platelet count (10^9/L)	n (%)	78 (69)	69 (85)	147 (76)
	Mean (SD)	380 (127)	380 (127)	380 (127)

Appendix 11

Number of participants who developed modified Beaugerie disabling disease (MBDD) over years from diagnosis, stratified by descriptors. CD = Crohn's disease.

**Descriptors**	**Years from diagnosis to developing MBDD**					**Total**
	**1**	**2**	**3**	**4**	**5**	
Hospitalisation due to a CD flare or complication	27	10	5	4	2	48
≥3 corticosteroid courses or dependence on corticosteroids	11	4	3	4	1	23
Intestinal resection >50 cm or surgery for perianal disease	1	0	0	0	0	1
Diarrhoea with nocturnal stools	0	0	0	1	0	1
Urgency	1	0	0	2	1	4
Abdominal pain due to intestinal obstruction	0	0	2	2	1	5
Fever	0	0	0	0	0	0
Fatigue	0	0	0	1	0	1
Joint pain not caused by another factor	1	1	0	0	1	3
Uveitis	2	0	0	0	0	2
Pyoderma gangrenosum	0	0	0	0	0	0
Total	43	15	10	14	6	88*

*Participants could fulfil multiple descriptors on the same day

**45 (23%) participants had an intestinal resection within 5 years of diagnosis.

MBDD = modified Beaugerie disabling disease

Data are n

Appendix 12

Modified Beaugerie disabling disease (MBDD) event free time, stratified by prespecified clinical predictors. Data are median (IQR).

**Prespecified clinical predictors**		**Developed MBDD**	**MBDD event free time (years)**
Age category (years)	<40	62	0.77 (0.42, 2.75)
	≥40	19	0.93 (0.39, 2.82)
Sex	Male	39	1.25 (0.41, 3.02)
	Female	42	0.68 (0.43, 2.11)
Smoking status	Non-smoker	49	0.93 (0.39, 2.69)
	Smoker	25	0.63 (0.49, 3.41)
Weight loss ≥5 kg prior to diagnosis	Absent	51	0.62 (0.41, 2.75)
	Present	18	1.75 (0.37, 3.02)
Initial need for steroid therapy	Absent	43	1.25 (0.52, 2.75)
	Present	38	0.50 (0.35, 2.82)
Developed MBDD ≤90 days from diagnosis	Absent	69	1.24 (0.46, 2.85)
	Present	12	0.51 (0.29, 1.21)
Perianal disease	Absent	70	0.88 (0.39, 2.55)
	Present	11	0.62 (0.46, 3.92)
Severe endoscopic disease	Absent	56	0.73 (0.41, 2.80)
	Present	20	0.88 (0.44, 2.02)
Disease behaviour	B1	50	1.03 (0.44, 2.85)
	B2	17	1.44 (0.47, 2.69)
	B3	14	0.47 (0.36, 1.77)
Location of disease behaviour	Ileocolonic	42	0.68 (0.43, 2.92)
	Ileal/Upper tract	28	1.51 (0.40, 2.33)
	Colonic	11	0.62 (0.39, 3.18)

Appendix 13

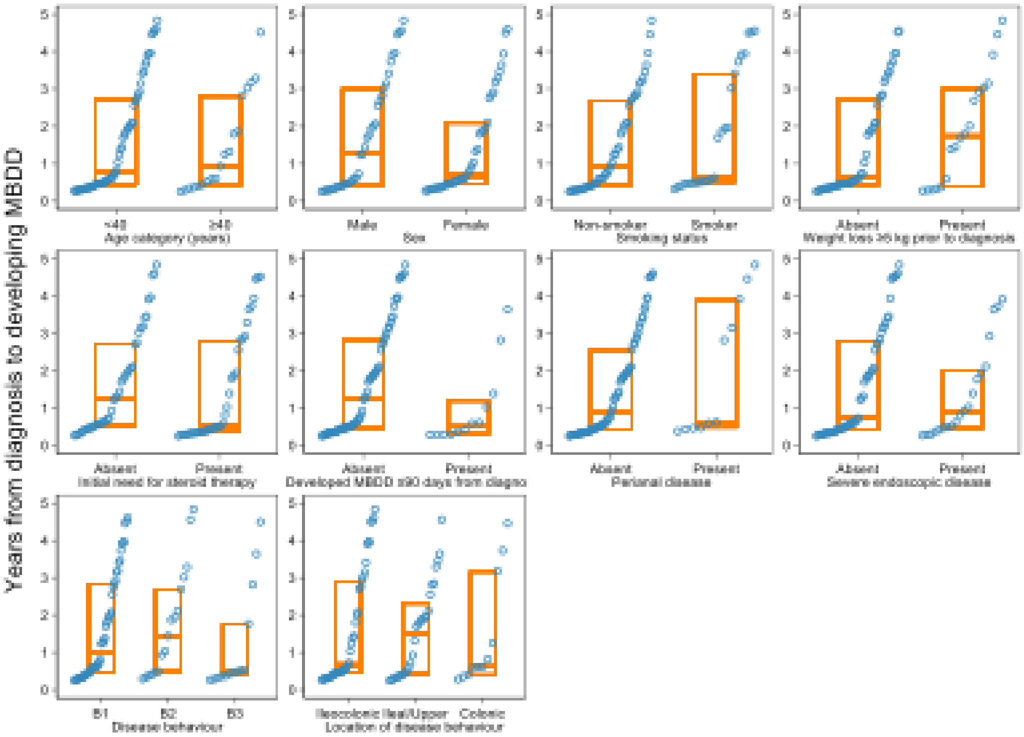

Scatter plots of years from diagnosis to developing modified Beaugerie disabling disease (MBDD), stratified by prespecified clinical predictors. Markers represent individual patients, and orange boxes represent median and IQR.

Appendix 14

Difference in sensitivity and specificity of prognostic models using Model B as the reference, stratified by risk group definition. For risk group definition 1, the high-risk group included the top 40% of participants with the greatest predicted risk from the model. For risk group definition 2, the high-risk group included participants with an absolute risk greater than or equal to 10%.

Prognostic model	Risk group definition	Sensitivity (95% CI)	Sensitivity difference (95% CI)	P-value	Specificity (95% CI)	Specificity difference (95% CI)	P-value
A	1	49 (39, 60)	-	-	66 (57, 74)	-	-
B1		51 (40, 61)	-1.2 (-4.9, 2.4)	>0.999	67 (58, 75)	0.9 (-1.7, 3.5)	>0.999
B2		52 (41, 62)	-2.5 (-9.6, 4.7)	0.688	68 (59, 76)	1.8 (-4.0, 7.5)	0.727
B3		51 (40, 61)	-1.2 (-6.7, 4.2)	>0.999	68 (59, 76)	1.8 (-1.5, 5.1)	0.500
C		53 (42, 64)	-3.7 (-10.3, 2.9)	0.375	70 (61, 78)	3.5 (-2.2, 9.3)	0.289
A	2	86 (77, 92)	-	-	35 (27, 45)	-	-
B1		91 (83, 96)	-4.9 (-10.9, 1.0)	0.125	29 (22, 38)	-6.2 (-11.5, -0.9)	0.016
B2		91 (83, 96)	-4.9 (-12.0, 2.1)	0.219	27 (20, 36)	-8.0 (-16.3, 0.3)	0.064
B3		91 (83, 96)	-4.9 (-10.9, 1.0)	0.125	29 (22, 38)	-6.2 (-12.2, -0.2)	0.039
C		91 (83, 96)	-4.9 (-12.0, 2.1)	0.219	32 (24, 41)	-3.5 (-9.3, 2.2)	0.289

Appendix 15

Variable loadings for principal components of prespecified predictors.

Prespecified predictors	Component 1	Component 2	Component 3	Component 4
Maximum mural thickness (MEGS)	0.36	-0.24	0.1	-0.13
Maximum mural T2 signal (oedema) (MEGS)	0.4	-0.14	0.05	-0.03
Maximum contrast enhancement pattern	0.31	-0.15	0.07	0.38
Maximum length of disease (MEGS)	0.38	-0.22	-0.01	-0.12
Abscess (MEGS)	0.25	0.56	0.19	0.09
Maximum fat stranding (sMARIA)	0.36	-0.13	0.1	0
Number of abnormal segments	0.33	-0.2	-0.11	-0.39
Maximum upper tract and small bowel stricturing	0.2	-0.05	-0.2	0.67
Maximum colon stricturing	0.03	0.12	0.89	-0.03
Maximum upper tract and small bowel penetrating	0.29	0.52	-0.15	0.18
Maximum colon penetrating	0.22	0.44	-0.27	-0.43

Appendix 16

Multivariable hazard ratios of prespecified clinical predictors for predicting development of MBDD within 5 years of diagnosis, using observed and imputed data (Model A).

Prespecified clinical predictors		Observed data (N=146)		Imputed data (N=194)	
		Hazard ratio (95% CI)	P-value	Hazard ratio (95% CI)	P-value
≥40 years of age		0.78 (0.40, 1.52)	0.466	0.73 (0.42, 1.27)	0.269
Female		0.87 (0.50, 1.50)	0.608	0.85 (0.54, 1.34)	0.485
Smoker		1.82 (1.07, 3.11)	0.028	1.50 (0.93, 2.42)	0.096
Weight loss ≥5 kg prior to diagnosis		0.79 (0.44, 1.43)	0.437	0.70 (0.38, 1.27)	0.240
Initial need for steroid therapy		2.42 (1.39, 4.21)	0.002	2.05 (1.28, 3.28)	0.003
Developed MBDD ≤90 days from diagnosis		1.18 (0.55, 2.56)	0.670	1.16 (0.59, 2.26)	0.664
Perianal disease		1.48 (0.65, 3.36)	0.346	1.22 (0.60, 2.47)	0.581
Severe endoscopic disease		0.73 (0.38, 1.41)	0.351	0.81 (0.45, 1.46)	0.492
Disease behaviour	B1	-	-		-
	B2	1.19 (0.62, 2.29)	0.607	1.33 (0.73, 2.43)	0.348
	B3	1.80 (0.86, 3.76)	0.119	1.40 (0.75, 2.63)	0.297
Location of disease behaviour	Ileocolonic	-	-		-
	Ileal/Upper tract	0.92 (0.51, 1.66)	0.773	0.89 (0.53, 1.49)	0.660
	Colonic	0.77 (0.32, 1.90)	0.575	0.98 (0.48, 1.99)	0.957

Appendix 17

Multivariable hazard ratios of prespecified clinical predictors for predicting development of MBDD within 5 years of diagnosis, using observed and imputed data (Model B1).

Prespecified predictors		Observed data (N=146)		Imputed data (N=194)	
		Hazard ratio (95% CI)	P-value	Hazard ratio (95% CI)	P-value
≥40 years of age		0.80 (0.41, 1.57)	0.519	0.73 (0.42, 1.28)	0.275
Female		0.87 (0.50, 1.51)	0.627	0.85 (0.54, 1.34)	0.486
Smoker		1.77 (1.02, 3.05)	0.041	1.49 (0.91, 2.44)	0.113
Weight loss ≥5 kg prior to diagnosis		0.77 (0.43, 1.41)	0.405	0.69 (0.37, 1.27)	0.232
Initial need for steroid therapy		2.44 (1.40, 4.27)	0.002	2.06 (1.28, 3.29)	0.003
Developed MBDD ≤90 days from diagnosis		1.09 (0.47, 2.53)	0.850	1.15 (0.58, 2.30)	0.690
Perianal disease		1.54 (0.67, 3.54)	0.307	1.23 (0.60, 2.52)	0.565
Severe endoscopic disease		0.71 (0.36, 1.37)	0.305	0.80 (0.43, 1.46)	0.462
Disease behaviour	B1	-	-		-
	B2	1.17 (0.60, 2.26)	0.644	1.33 (0.73, 2.44)	0.349
	B3	1.75 (0.83, 3.68)	0.142	1.40 (0.74, 2.64)	0.305
Location of disease behaviour	Ileocolonic	-	-		-
	Ileal/Upper tract	0.93 (0.51, 1.69)	0.818	0.89 (0.53, 1.49)	0.660
	Colonic	0.80 (0.32, 1.98)	0.632	0.99 (0.48, 2.01)	0.970
Normalised global MEGS (%)		1.01 (0.99, 1.03)	0.598	1.00 (0.98, 1.02)	0.918
Scores were normalised to enable comparison of the scores on a standardised scale					

Appendix 18

Multivariable hazard ratios of prespecified clinical predictors for prediction development of MBDD within 5 years of diagnosis, using observed and imputed data (Model B2).

Prespecified predictors		Observed data (N=146)		Imputed data (N=194)	
		Hazard ratio (95% CI)	P-value	Hazard ratio (95% CI)	P-value
≥40 years of age		0.74 (0.38, 1.46)	0.384	0.69 (0.39, 1.21)	0.193
Female		0.87 (0.50, 1.51)	0.628	0.86 (0.54, 1.37)	0.528
Smoker		1.95 (1.13, 3.37)	0.017	1.66 (1.01, 2.73)	0.046
Weight loss ≥5 kg prior to diagnosis		0.81 (0.45, 1.46)	0.479	0.69 (0.38, 1.26)	0.232
Initial need for steroid therapy		2.37 (1.37, 4.13)	0.002	2.02 (1.27, 3.23)	0.003
Developed MBDD ≤90 days from diagnosis		1.33 (0.60, 2.93)	0.483	1.25 (0.64, 2.44)	0.522
Perianal disease		1.30 (0.61, 3.20)	0.430	1.16 (0.57, 2.38)	0.676
Severe endoscopic disease		0.79 (0.40, 1.55)	0.493	0.91 (0.49, 1.68)	0.765
Disease behaviour	B1	-	-	-	-
	B2	1.18 (0.61, 2.28)	0.631	1.33 (0.73, 2.44)	0.349
	B3	1.93 (0.91, 4.09)	0.088	1.48 (0.78, 2.82)	0.232
Location of disease behaviour	Ileocolonic	-	-	-	-
	Ileal/Upper tract	0.86 (0.47, 1.57)	0.621	0.85 (0.51, 1.43)	0.547
	Colonic	0.75 (0.30, 1.84)	0.525	0.97 (0.48, 1.98)	0.934
Normalised global sMARIA (%)		0.99 (0.97, 1.01)	0.291	0.99 (0.97, 1.00)	0.153
Scores were normalised to enable comparison of the scores on a standardised scale					

Appendix 19

Multivariable hazard ratios of prespecified clinical predictors for predicting development of MBDD within 5 years of diagnosis, using observed and imputed data (Model B3).

Prespecified predictors		Observed data (N=146)		Imputed data (N=194)	
		Hazard ratio (95% CI)	P-value	Hazard ratio (95% CI)	P-value
≥40 years of age		0.77 (0.39, 1.50)	0.443	0.73 (0.42, 1.26)	0.257
Female		0.86 (0.50, 1.50)	0.601	0.85 (0.54, 1.34)	0.487
Smoker		1.87 (1.09, 3.22)	0.023	1.54 (0.94, 2.51)	0.085
Weight loss ≥5 kg prior to diagnosis		0.81 (0.45, 1.47)	0.496	0.70 (0.38, 1.28)	0.248
Initial need for steroid therapy		2.40 (1.38, 4.18)	0.002	2.05 (1.28, 3.27)	0.003
Developed MBDD ≤90 days from diagnosis		1.28 (0.57, 2.86)	0.555	1.19 (0.60, 2.32)	0.621
Perianal disease		1.42 (0.62, 3.27)	0.410	1.22 (0.60, 2.47)	0.588
Severe endoscopic disease		0.77 (0.39, 1.52)	0.451	0.84 (0.45, 1.55)	0.568
Disease behaviour	B1	-	-		-
	B2	1.18 (0.61, 2.28)	0.628	1.33 (0.73, 2.43)	0.349
	B3	1.88 (0.88, 3.98)	0.101	1.42 (0.75, 2.67)	0.281
Location of disease behaviour	Ileocolonic	-	-		-
	Ileal/Upper tract	0.89 (0.48, 1.62)	0.696	0.88 (0.53, 1.48)	0.635
	Colonic	0.76 (0.31, 1.86)	0.546	0.98 (0.48, 1.98)	0.945
Normalised Lémann index (%)		1.00 (0.98, 1.01)	0.567	0.98 (0.90, 1.07)	0.695
Scores were normalised to enable comparison of the scores on a standardised scale					

Appendix 20

Multivariable hazard ratios of prespecified clinical predictors for predicting development of MBDD within 5 years of diagnosis, using observed and imputed data (Model C).

**Prespecified predictors**		**Observed data (N=46)**		**Imputed data (N=194)**	
		**Hazard ratio (95% CI)**	**P-value**	**Hazard ratio (95% CI)**	**P-value**
≥40 years of age		0.62 (0.15, 2.49)	0.502	0.70 (0.38, 1.28)	0.248
Female		2.01 (0.55, 7.30)	0.290	0.92 (0.52, 1.62)	0.767
Smoker		3.81 (0.95, 15.31)	0.059	1.67 (0.91, 3.07)	0.099
Weight loss ≥5 kg prior to diagnosis		2.23 (0.69, 7.22)	0.181	0.72 (0.36, 1.45)	0.350
Initial need for steroid therapy		4.13 (1.24, 13.73)	0.021	2.00 (1.22, 3.31)	0.006
Developed MBDD ≤90 days from diagnosis		1.15 (0.20, 6.53)	0.875	1.22 (0.54, 2.75)	0.630
Perianal disease		4.73 (0.77, 29.19)	0.094	1.21 (0.56, 2.63)	0.630
Severe endoscopic disease		1.31 (0.30, 5.64)	0.721	0.83 (0.45, 1.55)	0.561
Disease behaviour	B1	-	-		-
	B2	1.18 (0.33, 4.18)	0.796	1.36 (0.71, 2.58)	0.353
	B3	3.26 (0.70, 15.20)	0.133	1.30 (0.61, 2.78)	0.499
Location of disease behaviour	Ileocolonic	-	-		-
	Ileal/Upper tract	1.38 (0.32, 5.99)	0.664	0.85 (0.49, 1.48)	0.555
	Colonic	1.96 (0.29, 13.31)	0.490	0.97 (0.46, 2.03)	0.933
CRP level (mg/L)		1.00 (0.98, 1.02)	0.791	1.00 (1.00, 1.01)	0.563
WBC count (10^9/L)		0.83 (0.62, 1.10)	0.188	0.98 (0.90, 1.06)	0.571
Faecal calprotectin level (µg/g)		1.00 (1.00, 1.00)	0.674	1.00 (1.00, 1.00)	0.929
Haemoglobin level (g/L)		1.03 (0.99, 1.08)	0.119	1.00 (0.98, 1.02)	0.991
Platelet count (10^9/L)		1.00 (1.00, 1.01)	0.101	1.00 (1.00, 1.00)	0.741

Appendix 21

Kaplan-Meier plots of the percentage of MBDD negative participants in low-risk and high-risk groups over years from diagnosis, stratified by risk group definition. For risk group definition 1, the high-risk group included the top 40% of participants with the greatest predicted risk from the model. For risk group definition 2, the high-risk group included participants with an absolute risk greater than or equal to 10%. (a) Model A and risk group definition 1, (b) Model A and risk group definition 2, (c) Model B1 and risk group definition 1, (d) Model B1 and risk group definition 2, (e) Model B2 and risk group definition 1, (f) Model B2 and risk group definition 2, (g) Model B3 and risk group definition 1, (h) Model B3 and risk group definition 2, (i) Model C and risk group definition 1, (j) Model C and risk group definition 2.

Blue lines represent the low-risk group and red lines represent the high-risk group.

Data are n.

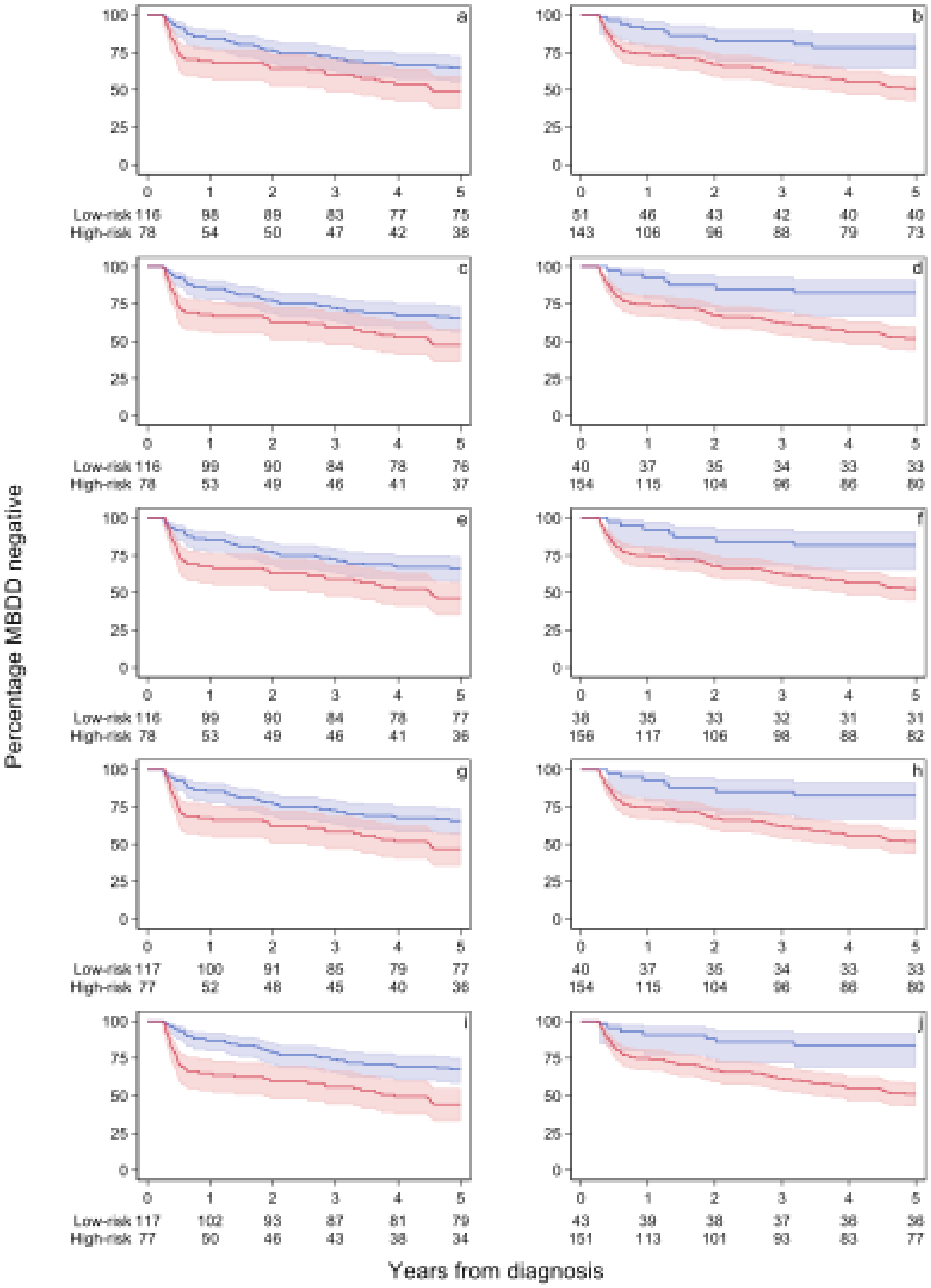
Percentage MBDD negative
Years from diagnosis
a
Low-risk 116 98 89 83 77 75
High-risk 78 54 50 47 42 38
b
51 46 43 42 40 40
143 106 96 88 79 73
c
Low-risk 116 99 90 84 78 76
High-risk 78 53 49 46 41 37
d
40 37 35 34 33 33
154 115 104 96 86 80
e
Low-risk 116 99 90 84 78 77
High-risk 78 53 49 46 41 36
f
38 35 33 32 31 31
156 117 106 98 88 82
g
Low-risk 117 100 91 86 79 77
High-risk 77 52 48 45 40 36
h
40 37 35 34 33 33
154 115 104 96 86 80
i
Low-risk 117 102 93 87 81 79
High-risk 77 50 46 43 38 34
j
43 39 38 37 36 36
151 113 101 93 83 77

Appendix 22

Number of participants correctly predicted to develop modified Beaugerie disabling disease (MBDD) within 5 years of diagnosis in a hypothetical sample of 1000 participants, stratified by risk group definition.

Prognostic model	Risk group definition	High-risk & developed MBDD (True-positive)	High-risk & did not develop MBDD (False-positive)	Low-risk & developed MBDD (False-negative)	Low-risk & did not develop MBDD (True-negative)
A	1	206	212	196	386
B1		212	206	191	391
B2		217	201	185	397
B3		212	206	185	397
C		222	196	175	407
A	2	361	57	376	206
B1		382	36	412	170
B2		382	36	422	160
B3		382	36	412	170
C		382	36	397	185

Appendix 23

Exploring the association between clinical and imaging variables with bowel resection within 5 years.

		No bowel resection 149 (77)	Bowel resection 45 (23)
Age category (years)	<40	104 (75)	34 (25)
	≥40	45 (80)	11 (20)
Sex	Male	76 (82)	17 (18)
	Female	73 (72)	28 (28)
Smoking status	Non-smoker	105 (81)	25 (19)
	Smoker	30 (64)	17 (36)
	Missing	14 (82)	3 (18)
Weight loss ≥5 kg prior to diagnosis	Absent	95 (78)	27 (22)
	Present	34 (74)	12 (26)
	Missing	20 (77)	6 (23)
Initial need for steroid therapy	Absent	98 (77)	29 (23)
	Present	51 (76)	16 (24)
Event ≤90 days from diagnosis	Absent	131 (78)	38 (22)
	Present	18 (72)	7 (28)
Perianal disease	Absent	130 (76)	40 (24)
	Present	18 (78)	5 (22)
	Missing	1 (100)	0 (0)
Severe endoscopic disease	Absent	102 (78)	28 (22)
	Present	33 (70)	14 (30)
	Missing	14 (82)	3 (18)
Disease behaviour	B1	118 (91)	12 (9)
	B2	16 (47)	18 (53)
	B3	15 (50)	15 (50)
	B2/B3	31 (48)	33 (52)
Location of disease behaviour	Ileocolonic	68 (72)	26 (28)
	Ileal/Upper	51 (74)	18 (26)
	Colonic	30 (97)	1 (3)
Perianal disease	Absent	130 (76)	40 (24)
	Present	18 (78)	5 (22)
	Missing	1 (100)	0 (0)
Maximum segmental sMARIA	0	24 (96)	1 (4)
	≥1	125 (74)	44 (26)
	<2	39 (98)	1 (2)
	≥2	110 (71)	44 (29)
Maximum segmental MEGS	<12	80 (91)	8 (9)
	≥12	69 (65)	37 (35)

Number of patients who had a resection within 5 years from diagnosis, stratified by clinical variables and disease activity scores at diagnosis.

Data are n (%).

	**Odds ratio (95% CI)**	**P-value**
≥40 years old	0.75 (0.35, 1.61)	0.456
Female	1.71 (0.87, 3.39)	0.122
Smoker	2.38 (1.14, 4.98)	**0.021**
Weight loss ≥5 kg prior to diagnosis	1.24 (0.57, 2.72)	0.589
Initial need for steroid therapy	1.06 (0.53, 2.13)	0.870
Event ≤90 days from diagnosis	1.34 (0.52, 3.45)	0.543
Perianal disease	0.90 (0.32, 2.59)	0.849
Severe endoscopic disease	1.55 (0.73, 3.28)	0.257
B2/B3 disease	10.47 (4.85, 22.61)	**<0.001**
B3 disease	4.47 (1.97, 10.12)	**<0.001**
Max segmental sMARIA ≥1	8.45 (1.11, 64.30)	**0.039**
Max segmental sMARIA ≥2	15.60 (2.08, 117.07)	**0.008**
Max segmental MEGS ≥12	5.36 (2.34, 12.29)	**<0.001**

Univariable logistic regression with dependent variable coded as 0 = had no resection and 1 = had a resection.

Appendix 24

Number of participants who started biologic therapy <180 days from diagnosis and developed MBDD ≥90 days later, stratified by maximum segmental sMARIA score.

MBDD = modified Beaugerie disabling disease, sMARA = simplified magnetic resonance index of activity

	Maximum segmental sMARIA				Global sMARIA		Total
	<1 N=19	≥1 N=139	<2 N=31	≥2 N=127	<6 N=98	≥6 N=60	N=158
Did not start biologic therapy & developed MBDD	4 (21)	19 (14)	5 (16)	18 (14)	17 (17)	6 (10)	23 (15)
Did not start biologic therapy & did not develop MBDD	12 (63)	51 (37)	22 (71)	41 (32)	40 (41)	23 (38)	63 (40)
Started biologic therapy <180 days from diagnosis & developed MBDD ≥90 days later	1 (5)	15 (11)	1 (3)	15 (12)	10 (10)	6 (10)	16 (10)
Started biologic therapy <180 days from diagnosis & did not develop MBDD	0 (0)	23 (17)	1 (3)	22 (17)	12 (12)	11 (18)	23 (15)
Started biologic therapy ≥180 days from diagnosis & developed MBDD ≥90 days later	0 (0)	6 (4)	0 (0)	6 (5)	5 (5)	1 (2)	6 (4)
Started biologic therapy ≥180 days from diagnosis & did not develop MBDD	2 (11)	25 (18)	2 (6)	25 (20)	14 (14)	13 (22)	27 (17)
